# Optimization of Thermoreversible* In Situ* Nasal Gel of Timolol Maleate

**DOI:** 10.1155/2016/6401267

**Published:** 2016-05-16

**Authors:** Swati Jagdale, Nirupama Shewale, Bhanudas S. Kuchekar

**Affiliations:** MAEER's Maharashtra Institute of Pharmacy, Savitribai Phule Pune University, MIT Campus, S. No. 124, Kothrud, Pune 411038, India

## Abstract

Nasal route had shown better systemic bioavailability due to its large surface area, porous endothelial membrane, high total blood flow, and avoidance of first-pass metabolism. Timolol maleate is a beta blocker used primarily in the treatment of hypertension. Drug undergoes extensive hepatic first-pass metabolism (80%). The drug has half-life of 4 hrs. Oral bioavailability of timolol maleate is 61%. The aim of the present study was to optimize controlled release* in situ* nasal delivery for timolol maleate. HPMC and Poloxamer 407 were selected as polymer in formulation of thermoreversible* in situ* nasal gel. Optimization was carried out using 3^2^ factorial design. It was observed that formulations f1 and f4 revealed the highest % drug release, that is, 93.57% and 91.66%, respectively. Factorial design study indicated that the drug release and viscosity were most significant dependent factors.* Ex vivo* diffusion study through nasal mucosa indicated 67.26 ± 2.10% and 61.07 ± 2.49% drug release for f1 and f4 formulations. f1 was the optimized batch. This batch thus can act as a potential nasal delivery with enhanced bioavailability for the drug.

## 1. Introduction

Hypertension is a major health problem throughout the world because of its high prevalence and association with increased risk of cardiovascular diseases. It is estimated that more than one billion people are suffering from hypertension worldwide [1, 2]. In recent years many drugs have been shown to achieve better systemic bioavailability through nasal route. This is due to the large surface area, porous endothelial membrane, high total blood flow, avoidance of first-pass metabolism, and ready accessibility of the route [3].

The nasal cavity is covered by a thin mucosa which is well vascularised. The absorbed drug from the nasal cavity will pass through the mucus layer which is the first step in absorption. A drug molecule can be transferred quickly across the single epithelial cell layer directly to the systemic blood circulation without first-pass hepatic and intestinal metabolism. The effect is often reached within five minutes for a smaller drug molecule. Nasal administration thus is used as an alternative route to oral administration especially for those drugs which extensively get degraded in the gut/liver and for drugs having poor absorptivity [4, 5].

*In situ* polymeric formulations are the drug delivery system that are in solution form before administration in the body but once administered undergo* in situ* gelation [6, 7]. The approaches used for formulation of* in situ* gelling system include physiological stimuli, osmotic stimuli, chemical stimuli, pH-triggered system, and temperature dependent system. Temperature is most widely used stimulus in environmentally responsive polymer system. The change in temperature not only is relatively easy to control but also has easy applicability both* in vitro* and* in vivo*. In these systems, gelling of the solution is triggered by change in temperature. These hydrogels are liquid at room temperature (20–25°C) and undergo gelation with increase in temperature when in contact with body fluid (35–37°C). The polymers which show temperature induced gelation include chitosan, pluronics, tetronics, xyloglucans, and hydroxypropyl methyl cellulose or hypromellose [8–10].

Timolol maleate is a beta blocker used primarily in the treatment of hypertension. Drug undergoes extensive hepatic first-pass metabolism (80%) via cytochrome P450 2D6 isoenzyme. The drug has half-life of 4 hrs. Oral bioavailability of timolol maleate is 61% and 75% for intravenous route. Maximum blood plasma concentrations ranging from 10 ng/mL to 100 ng/mL are attained within 1 to 2.4 hours after either acute or chronic administration of 2.5 mg to 20 mg of drug twice daily. The effect of food on the rate and extent of oral absorption of drug is not significant.

Literature survey had indicated that for timolol maleate work has been carried out in area of matrix tablet in 2014, ocular insert in 2012, and mucoadhesive buccal tablet in 2010 [11–13]. The market survey indicated presence of ophthalmic gel for the drug. It gives the challenge to design a drug delivery to overcome the problems associated with the first-pass effect and bioavailability and to give a controlled drug release in treatment of hypertension. The aim of the present work was to optimize* in situ* nasal drug delivery. Experiments were designed first with the preformulation study using different gelling agent as HPMC, sodium alginate, Poloxamer 407, and locust bean gum. Factorial design was used as an experimental tool with polymers HPMC and Poloxamer 407 for optimization. The final formulation is expected to form* in situ* gel in nasal cavity. Gelation temperature and mucoadhesive strength therefore were first most important parameter for evaluation. The second most important parameter was % drug release (*in vitro* as well as* ex vivo*) to meet the objective of giving a controlled release of drug at the site of action reducing the dosing frequency of drug [14–16].

## 2. Material and Method

Timolol maleate was a gift sample from Micro Lab Pvt. Ltd., Bangalore. HPMC was a gift sample from Colorcon Asia Pvt. Ltd., Mumbai. Poloxamer 407 was purchased from Analab Mumbai.

### 2.1. Characterization of Timolol Maleate

The drug sample was analyzed for the organoleptic properties as color, odour, and appearance. Melting point was determined by capillary method.

### 2.2. UV Spectroscopic Study and Validation of UV Spectrophotometric Method

Timolol maleate was dissolved in phosphate buffer pH 6.4. Solution (10 *µ*g/mL) was scanned in the range of 200–400 nm and the spectrum was recorded. Linearity, accuracy (recovery test), precision, robustness, ruggedness, LOD, and LOQ were determined (ICH).

### 2.3. FTIR Spectroscopy

FTIR spectrum of timolol maleate was recorded using Fourier transform infrared spectrophotometer (Jasco 4600 IR) with diffuse reflectance principle with KBr. The spectrum was scanned over a frequency range of 4000–400 cm^−1^.

### 2.4. Drug-Polymer Interaction Study

Samples of drug and polymer alone as well as in combination (1 : 1 ratio) were placed in stability chamber for one month at 40°C ± 2°C/75% RH ± 5% RH. FTIR and UV spectra for samples were recorded.

### 2.5. Differential Scanning Calorimeter (DSC)

DSC measurements were performed on a differential scanning calorimeter (DSC-60, Shimadzu Corporation, Japan). Inert atmosphere was maintained by purging nitrogen gas at a flow rate of 50 mL/min. Accurately weighed samples (about 5–10 mg) were placed in a sealed aluminum pan and the samples were heated under nitrogen gas flow (20 mL/min) at a scanning rate of 10°C per min from 40 to 340°C. An empty aluminum pan was used as reference.

### 2.6. Formulation Study

#### 2.6.1. Preliminary Batches

In the beginning, HPMC, Poloxamer 407, sodium alginate, and locust bean gum were tried in concentrations ranging from 0.2 to 2% individually. Sodium alginate with locust bean gum and Poloxamer were tried in combination. Based on the gelation property the finalized preliminary trial batches are as shown in Table 1.

#### 2.6.2. Preparation of* In Situ* Nasal Gel

*In situ* nasal gel was prepared by cold technique. To the solution of drug in distilled water, HPMC was added and stirred continuously. After this Poloxamer 407 was added with continuous stirring. The formulation was kept at 4°C overnight until the clear gel was obtained [17–20].

### 2.7. Design of Experiment (DOE)

Based on the results obtained from preliminary batches experimental runs for optimization with the help of 3^2^ factorial design were designed. Two independent variables as concentration of HPMC and Poloxamer 407 were fixed as shown in Table 2 [21]. The formulations were prepared as shown in Table 3.

Evaluation of gel was carried out for the following parameters [17–20].

#### 2.7.1. Appearance

The clarity was determined by visual inspection under black and white background.

#### 2.7.2. pH

Formulation equivalent to 10 mg of drug was transferred and diluted with distilled water. pH of resulting solution was determined using pH meter.

#### 2.7.3. Drug Content

Formulation equivalent to 10 mg of drug was diluted with distilled water and after suitable dilutions the absorbance was measured at 294 nm using UV visible spectrophotometer. The drug concentration was calculated as shown in (1) and (2):

(1) $$\begin{array}{c} X=Y-\frac{C}{M}, \end{array}$$

where *X* is conc. in *µ*g/mL, *Y* is absorbance of solution at 294 nm, *C* is intercept of standard curve, and *M* is slope of standard curve.

Further,  % drug content was calculated from the conc. using the following equation:

(2) %  Drug  content=Concentration  of  drug  in  sample  solutionEquivalent  conc.  of  drug  taken×100.

#### 2.7.4. Gelation Temperature

Formulation equivalent to 10 mg was transferred to a test tube and immersed in a water bath. The temperature of water bath was increased slowly and left to equilibrate for 5 min at each new setting. The sample was then examined for gelation, which was said to have occurred when the meniscus would no longer move upon tilting through 90°C [6].

#### 2.7.5. Mucoadhesive Strength

Mucoadhesive potential of each formulation was determined by measuring a force required to detach the formulation from membrane. It was measured by modified balance. Egg membrane was used for the study. It was mounted on lower side of glass surface using adhesive tape while another membrane was fixed on upper side of glass slide, kept on inverted cylinder. Gel equivalent to the 10 mg was placed on membrane surface. Empty beaker was attached to another side of the balance. Membrane surface with gel formulation and upper membrane surface were held in contact with each other for 2 min to ensure intimate contact. Water was added to the beaker until detachment takes place. The mucoadhesive force was expressed as the detachment stress in dynes/cm^2^ as shown in [7]

(3) $$\begin{array}{c} Mucoadhesive\;\;strength\left(\;dynes/{cm}^{2}\;\right)=\frac{mg}{A}, \end{array}$$

where *m* is weight required for detachment in gram, *g* is acceleration due to gravity (980 cm/s^2^), and *A* is area of mucosa exposed.

#### 2.7.6. Viscosity

Viscosity was determined using Brookfield viscometer [22].

#### 2.7.7. *In Vitro* Drug Release Study

(*1) In Vitro Diffusion Study: Cellophane Membrane.* Franz diffusion cell was used for permeation study with cellophane membrane (mol.wt. 12,000–14,000) having pore size of 2.4 nm. Cellophane membrane was placed in between the donor and the receptor compartment. Gel containing drug equivalent to 10 mg was applied on surface of membrane. It was in contact with receptor compartment containing 25 mL of phosphate buffer pH 6.4. The cell was agitated by a magnetic stirrer at 50 rpm and maintained at 37°C. Aliquots were withdrawn at intervals till 480 min and were replaced with equal volume of fresh phosphate buffer pH 6.4. Absorbance was measured at 294 nm.

(*2) In Vitro Diffusion Study: Egg Membrane.* Instead of cellophane membrane, egg membrane was used and same procedure was followed as given in Section 2.7.7(1).

(*3) Ex Vivo Diffusion Study (Nasal Mucosa). Ex vivo* drug permeation study was carried out for best formulations. Fresh nasal tissue was removed from nasal cavity of sheep obtained from local slaughter house. Mucosa was stored in saline water at frozen condition. It was placed in between the donor and the receptor compartment. Same procedure as given in Section 2.7.7(1) was followed [19, 20].

(*4) Release Experiment*

*Model Dependent Method.* In order to have insight into the drug release mechanism from* in situ* nasal gel, drug release data were examined to find out order as zero-order, first-order, and Higuchi model, Hixson and Crowell model, and Korsmeyer-Peppas model. The equation for the models is as follows:

(4) (i)  Zero  order:  Ft=K0t(ii)  First  order:  ln⁡Ft=ln⁡F0+Kt(iii)  Higuchi  matrix:  Ft=K√t(iv)  Hixson  and  Crowell:  F=1001−1−Kt3(v)  Korsmeyer-Peppas  model:  Ft=Ktn,

where *F* is fraction of drug release, *K* is constant time, and *n* is diffusion coefficient.

#### 2.7.8. Histopathological Study

In histopathological evaluation, fresh nasal tissue was removed from the nasal cavity of sheep. The tissue was inserted in the diffusion cell. Phosphate buffer pH 6.4 was then added to the receptor chamber. Gel was applied to the mucosa and left for 24 hr. After 24 hrs each piece of mucosa was carefully removed from the diffusion chamber and rinsed with phosphate buffer. Sections were examined under a light microscope, to detect any damage to the tissue.

#### 2.7.9. Optical Microscopy

Optical microscopy for gel was carried out by using Motic digital microscope. The sample was spread on the glass slide. This slide was focused under 10x magnification lenses and the pictures were captured.

#### 2.7.10. Flux

The flux was calculated by Fick's law of diffusion [23]. Flux is defined as the amount of the drug passing through a unit cross sectional area *S* in unit time *t*. PCP disso V3 software was used for calculation of flux. The equation used for calculating the flux is

(5) $$\begin{array}{c} J=\frac{dm}{S}\cdot dt, \end{array}$$

where *m* is amount of drug passing through the mucosa, *S* is surface area of nasal mucosa, and *t* is total time of diffusion through the nasal mucosa.

## 3. Results and Discussion

### 3.1. Characterization of Timolol Maleate

Melting point of timolol maleate was observed at 203°C. The *λ*_max_ of timolol maleate was observed at 294 nm.

### 3.2. UV Spectroscopic Study and Validation of UV Spectrophotometric Method

Normal physiological pH of the nasal mucosa ranges from 4.5 to 6.5. Therefore, phosphate buffer pH 6.4 was prior choice as analytical medium for present work.

#### 3.2.1. Linearity

Timolol maleate exhibited maximum absorption at 294 nm and obeyed Beer's law in the range of 4–24 *µ*g/mL. Linear regression of absorbance versus concentration gave equation

(6) $$\begin{array}{c} y=0.0213x-0.033 \end{array}$$

with a correlation coefficient of 0.996.

#### 3.2.2. Accuracy (Recovery Test)

The accuracy of the method was expressed as the percentage recovery of timolol maleate and was 98–102% indicating that there was no interference by the solvent.

#### 3.2.3. Intraday Precision

The relative standard deviation (RSD) for intraday precision was less than 2%.

#### 3.2.4. Repeatability

RSD value for repeatability was less than 2%.

#### 3.2.5. Robustness

No significant difference was observed in the results demonstrating that the method is robust. RSD was less than 2%.

#### 3.2.6. Ruggedness

Ruggedness of proposed method was determined by the analysis of sample solutions (8 *µ*g/mL) at different wavelength.

#### 3.2.7. Detection and Quantitation Limit

LOD was found to be 0.708 and LOQ was found to be 0.343.

It was concluded that the proposed method was accurate, linear, precise, robust, simple, and sensitive.

### 3.3. FTIR Spectroscopy

**Figure 1:**
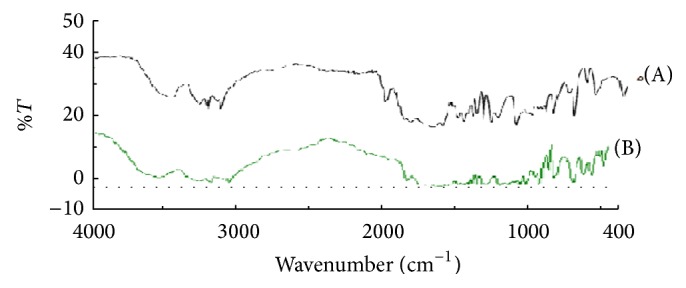
Infrared spectrum of (A) standard timolol maleate (IP) and (B) sample of timolol maleate.

The infrared spectrum is very specific for each chemical structure, since small structural differences result in significant spectral changes. FTIR spectrum is characteristic of entire molecule and provides structural information by referring to peaks associated with characteristics groups. Timolol maleate exhibited characteristic peaks as shown in Figure 1. The values reported are as indicated in Table 4. Reported values match with the standard values given in Indian pharmacopoeia, confirming the purity of the drug [11].

### 3.4. Drug-Polymer Interaction Study

**Figure 2:**
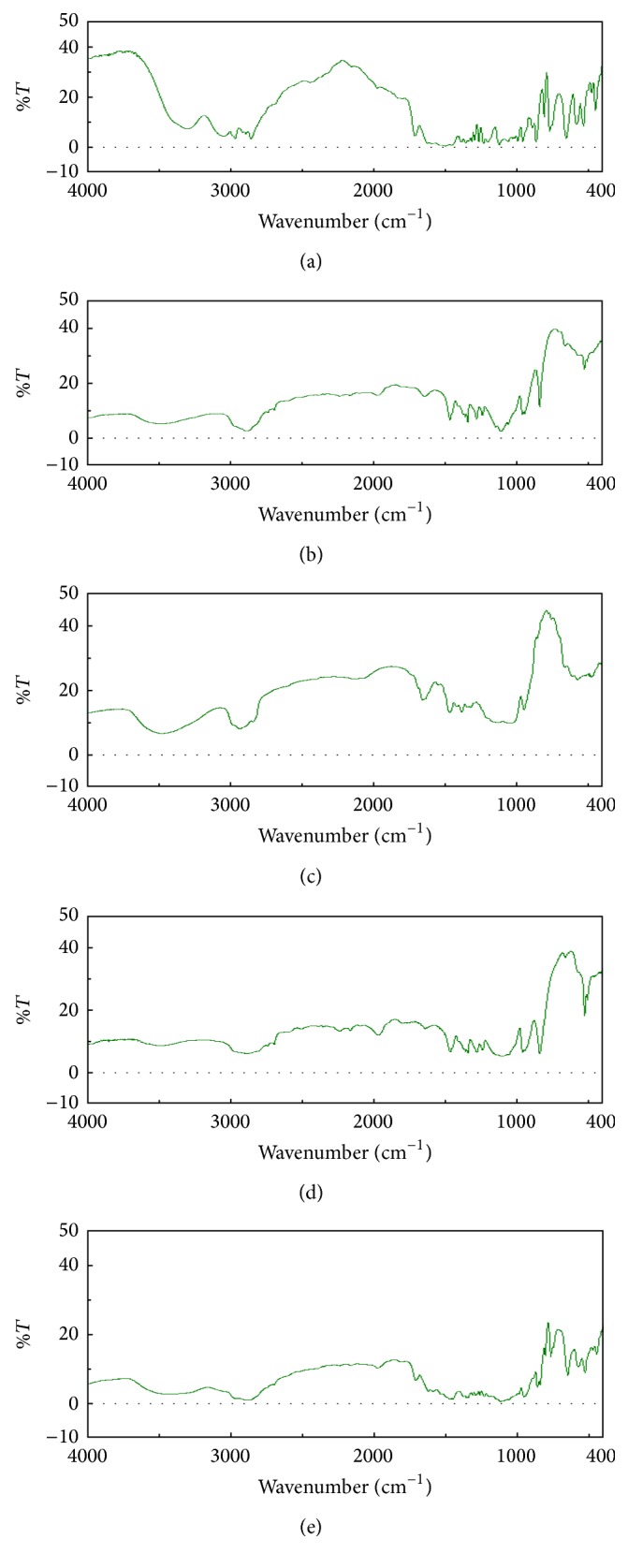
IR spectra of (a) timolol maleate, (b) HPMC + PF 27, (c) HPMC, (d) PF 127, and (e) timolol maleate + HPMC + PF 127.

From Figure 2, it was observed that there was no significant change in the peak values of drug when compared with the standard values as indicated in Table 4. This indicated absence of any chemical reaction between drug and polymer.

### 3.5. Differential Scanning Calorimetric Analysis

**Figure 3:**
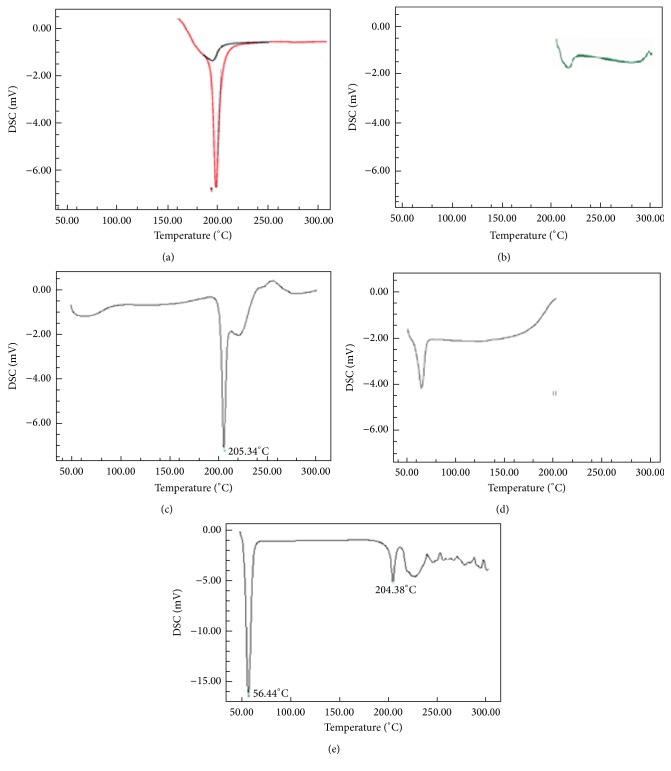
(a) DSC thermogram of pure drug, (b) pure HPMC, (c) drug + HPMC, (d) Poloxamer 407, and (e) drug + Poloxamer 407.

DSC spectrum as shown in Figure 3(a) indicated melting point of timolol maleate sharp at 205°C.  Figure 3(b) indicated melting point of HPMC at 56°C. Drug with HPMC (Figure 3(c)) had shown sharp peak at 205.30°C and 56.3°C. Poloxamer 407 (Figure 3(d)) showed sharp peak at 56.8°C. Drug with Poloxamer 407 (Figure 3(e)) showed sharp peak at 204.38°C and 56.44°C, respectively. From this study it can be concluded that the peak of drug remains unchanged in combination with the polymer indicating absence of any chemical interaction between drug and polymer.

### 3.6. Preliminary Batches

All the preliminary batches were evaluated for the pH and gelation temperature. Batches with locust bean gum and sodium alginate failed to get solubilized and hence were not further accepted for study. 0.4% HPMC and 42% Poloxamer 407, 0.6% HPMC and 44% Poloxamer 407, and 0.6% HPMC and 45% Poloxamer 407 were tried at initial stage. All these batches failed in gelation criteria. 0.8% HPMC and 46% Poloxamer 407 had shown good result by gelation at 36.5°C and also shown 93.67% drug release at 8 hrs. Based on this result factorial design was finalized. It was found that with increase in concentration of HPMC and Poloxamer 407 good gelation was observed. This system also maintains pH in the range.

### 3.7. Design of Experiment (DOE)

#### 3.7.1. Appearance

The appearance of formulations was found to be clear.

#### 3.7.2. pH

It is known that the normal physiological pH of nasal mucosa is 4.5–6.5. However, the nasal mucosa can tolerate solutions within pH range of 3–10. pH for all formulation was found in range of 4.7–6.9.

#### 3.7.3. Drug Content

Drug contents were found in the range of 84.42–96.66%. It was observed that f5 showed less drug content, that is, 84.42%, and f3 showed high drug content, that is, 96.66%.

#### 3.7.4. Gelation Temperature

Gelation temperature range suitable for nasal gel is 32–35°C. Gelation temperature for all formulations was found in the range of 36–31°C. It was observed that as the concentration of Poloxamer 407 and HPMC increases there was decrease in gelation temperature.

#### 3.7.5. Mucoadhesive Strength

Mucoadhesive strength was determined by measuring the force required to detach the formulation from membrane, that is, detachment force. It was observed that HPMC and Poloxamer 407 both had shown effect on mucoadhesive strength. As the concentration for both polymers increases, mucoadhesive strength was also found to be increased. f1 had shown least mucoadhesive strength, that is, 1172.42, and f9 had showed the highest mucoadhesive strength, that is, 2483.62 (as shown in Table 5).

#### 3.7.6. Viscosity Measurement

From the results it was observed that HPMC had viscosity enhancing effect. The viscosity was in range of 14450 cps to 25900 cps. With increase in concentration of HPMC, viscosity was found to be increasing with retarding % drug release. Residence time got enhanced with higher viscosity, but drug absorption was found to be diminished. Profile of viscosity followed shear thickening pattern indicating sol to gel conversion.

#### 3.7.7. *In Vitro* Drug Diffusion Study

**Figure 4:**
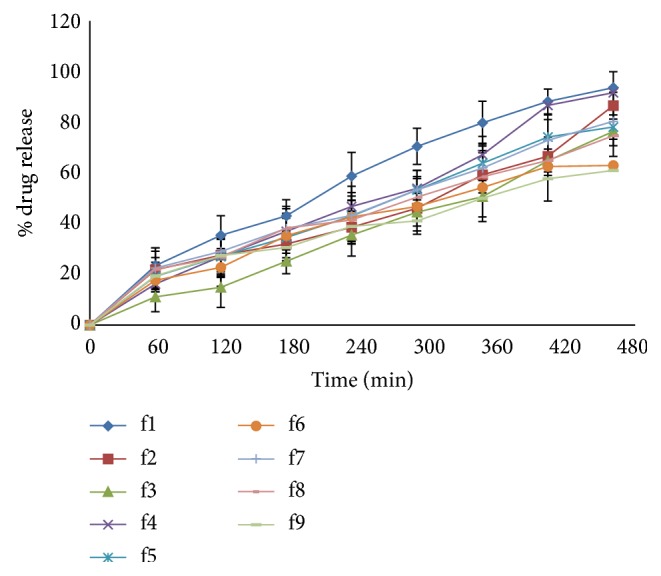
% drug release for formulations f1–f9.

(*1) Cellophane Membrane.* From Figure 4, it was observed that polymer concentration affects drug release. As the concentration of HPMC and Poloxamer 407 increases, it retarded % drug release. % drug release for batches f1 and f4 was found as 93.57% and 91.66%, respectively.

(*2) Kinetic Study and Mechanism of Drug Release.* Korsmeyer-Peppas was found as best fit model for batches f1 and f4.

### 3.8. Experimental Design

The data obtained was treated using design-expert 9.01.3 software and analyzed statistically using analysis of variance (ANOVA). DOE indicated no interaction between two independent variables. The data was further selected on 3D response surface methodology to study the interaction of *X*_1_ and *X*_2_ on dependent variables.

#### 3.8.1. Effect of Formulation Variables on Drug Release at 480 min

The regression equation obtained for % drug release at 480 min is as follows:

(7) Drug  release  at  480 min=−185.24−17.49A+14.089B,

where *A* is HPMC conc. and *B* is Poloxamer 407.

The model terms were found to be significant with value of *R* ^2^ 0.9717, which indicated the adequate fitting to linear model. Values of “prob > *F*” less than 0.05 confirmed that the model terms were significant. Also, the “pred *R*-squared” value of 0.8152 was in reasonable agreement with the “adj *R*-squared” value of 0.9575, as it can be seen from Table 6 and (7). The “adequate precision” measures the signal to noise ratio. A ratio greater than 4 is desirable. Present work ratio was 25.552 indicating adequate signal. This inferred that the model can be used to navigate the design space.

**Figure 5:**
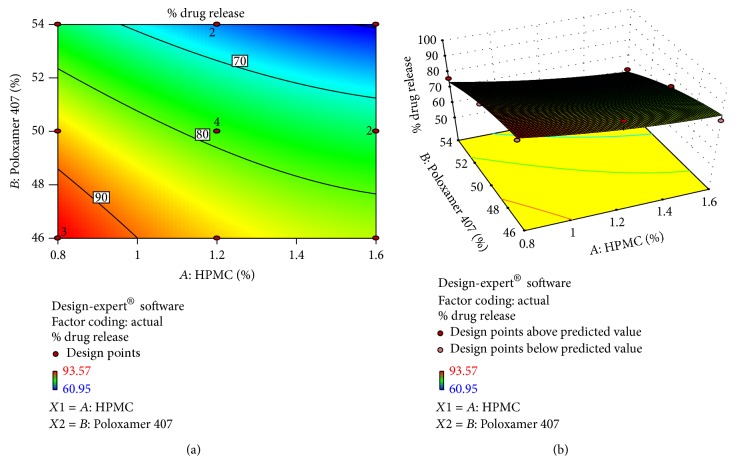
(a) Contour plot. (b) Response surface plot showed relationship in between % drug release at 480 min and polymer conc.

A positive sign of the coefficient indicated a synergistic effect while a negative sign indicated an antagonistic effect on the response. The larger coefficient means the independent variable has more potent influence on the response. Overall it was concluded that HPMC and Poloxamer 407 caused significant change in the response. As the concentration of HPMC increases, there was decrease in % drug release found (Figure 5).

#### 3.8.2. Effect of Formulation Variables on Gelation Temperature

The regression equation obtained for the gelation temperature is as follows:

(8) $$\begin{array}{c} Gelation\;\;temperature=+144.74-7.24A-3.899B, \end{array}$$

where *A* is HPMC conc. and *B* is Poloxamer 407.

The model terms for the gelation temperature were found to be significant with value of *R* ^2^ 0.9734, which indicated the adequate fitting to linear model. Values of “prob > *F*” less than 0.05 confirmed that the model terms were significant. Also, the “pred *R*-squared” value of 0.8281 was in reasonable agreement with the “adj *R*-squared” value of 0.9601, as it can be seen in Table 6.

The “adequate precision” measures the signal to noise ratio. A ratio greater than 4 is desirable. Present work ratio was 24.75 indicating an adequate signal. This inferred that the model can be used to navigate the design space.

**Figure 6:**
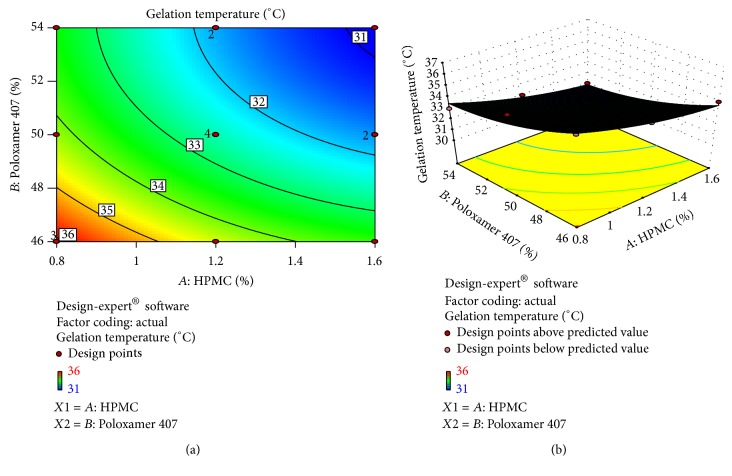
(a) Contour plot. (b) Response surface plot showed relationship in between % gelation temperature and polymer conc.

The graph reveals the contribution of HPMC and Poloxamer 407 to gelation temperature. Signs of HPMC and Poloxamer 407 both are negative indicating that both polymers have inverse relation with the gelation temperature. As the concentration of HPMC and Poloxamer 407 increased, the gelation temperature was decreased. Overall the concentration of polymer affected the response significantly as indicated by (8). It was observed that higher concentration of polymer decreases the gelation temperature (Figure 6).

### 3.9. Validation of Statistical Model

After statistical analysis by design-expert software f1 was found as optimized batch. The experimental values for the % drug release at 480 min and gelation temperature were found very close to the predicted values by the software as indicated in Table 7. Gelation temperature was almost similar to minimum standard deviation. f1 batch indicated as optimized batch proved that the model was successfully validated.

### 3.10. *In Vitro* Drug Diffusion Study (Egg Membrane)

**Figure 7:**
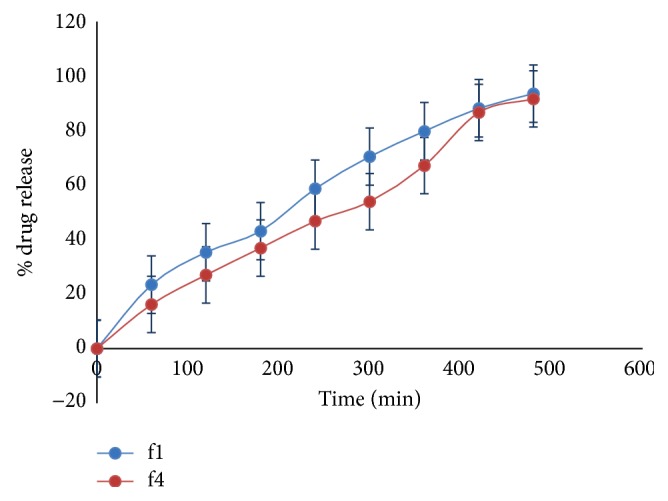
% drug release of formulations f1 and f4.

f1 was first option given as optimized by the software and f4 was second option. From Figure 7, it was found that f1 and f4 have shown % drug release, 84.40% and 80.95%, respectively.

### 3.11. *Ex Vivo* Drug Permeation Study

**Figure 8:**
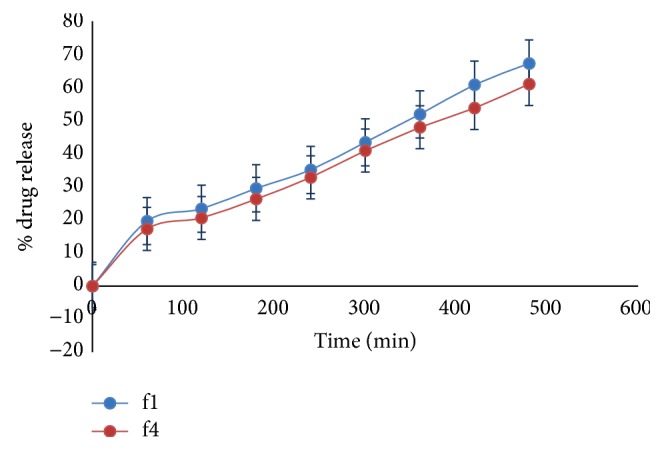
*Ex vivo* % drug release of formulations f1 and f4.

Study has been carried out on f1 and f4 batches. From Figure 8, it was observed that, as compared to cellophane membrane and egg membrane, nasal mucosa gave less % drug release. The reason may be attributed to high thickness of mucosa. f1 and f4 have shown % drug release, 67.26%  and 61.07%, respectively.

### 3.12. Optical Microscopy

**Figure 9:**
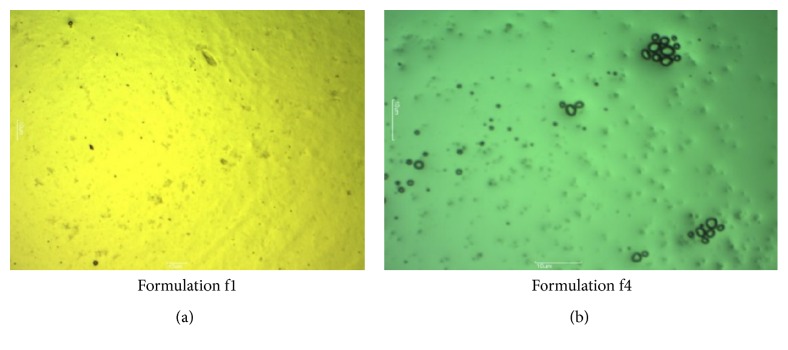
Optical microscopic images of formulation at 4x magnification.

**Figure 10:**
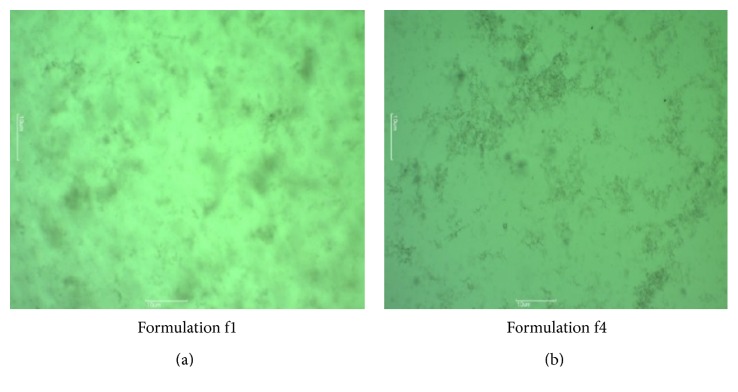
Optical microscopic images of formulation at 10x magnification.

Morphology of gel was studied under light microscope. The gel was evenly spread on glass slide and cover slip was placed over it and then it was mounted and viewed by light microscope under magnifications 4x and 10x. As seen in Figures 9 and 10, it was observed that drug was uniformly distributed in formulation f1 [24].

### 3.13. Histopathological Study

**Figure 11:**
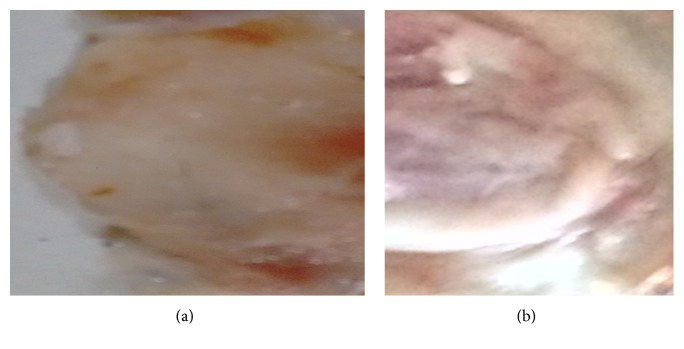
(a) Controlled mucosa treated with phosphate buffer pH 6.4. (b) Mucosa treated with formulation f1.

In histopathological study it was found that there are no changes to the nasal mucosa after the study. The microscopic observation indicated that optimized formulation f1 has no effect on the nasal mucosa. The epithelial layer was intact and showed no damage (Figure 11) to the mucosa. Thus, gel formulations seem to be safe with respect to nasal administration [25].

### 3.14. Stability Study

Stability study of optimized formulation f1 was studied at 30°C ± 2°C/65% RH ± 5% RH and 40°C ± 2°C/75% RH ± 5% RH for three months for pH and % drug content. Both the parameters indicated no significant difference from the initial value which proved stability for f1 batch [26].

### 3.15. Flux

Flux for the drug through different membrane (area of 0.8 cm^2^) was calculated. Comparative study of average flux of cellophane membrane, egg membrane, and nasal mucosa has been carried out and results are as shown in Table 8. Formulation had shown good flux properties indicating good permeability across the nasal mucosa.

## 4. Conclusion

HPMC and Poloxamer 407 were used as independent variables in factorial design for optimization of* in situ* controlled release nasal gel for timolol maleate. f1 and f4 gave significant and good results with respect to gelation temperature, mucoadhesive strength, and release study. f1 and f4 revealed the highest drug release through the egg membrane, that is, 84.40 ± 2.54% and 80.95 ± 2.45%, respectively. Statistical optimization gave f1 as optimized batch. The microscopic observations indicate that f1 had no significant effect on the nasal mucosa. In conclusion, it can be said that a stable, effective* in situ* nasal gel of timolol maleate was formulated which will bypass the first-pass effect, improve the bioavailability, and give a controlled release for the drug at the site which gave possibility of lowering the dosing frequency.

## Figures and Tables

**Table 1:** Preliminary batches.

Batch code	HPMC (% w/w)	Poloxamer 407 (% w/w)
1	0.4	42
2	0.6	44
3	0.6	45
4	0.8	46
5	1.6	54
6	1.8	55

**Table 2:** Coded level for variable (f1–f9).

Independent variables	Coded levels		
	−1	0	+1
HPMC conc. (% w/w)	0.8%	1.2%	1.6%
Poloxamer 407 conc. (% w/w)	46%	50%	54%

**Table 3:** Formulation of factorial batches.

Sr. number	Ingredient (% w/w)	f1	f2	f3	f4	f5	f6	f7	f8	f9
1	Timolol maleate	1.40	1.418	1.470	1.438	1.449	1.464	1.478	1.484	1.492
2	Poloxamer 407	46	50	54	46	50	54	46	50	54
3	HPMC	0.8	0.8	0.8	1.2	1.2	1.2	1.6	1.6	1.6
4	Distilled water	q.s. up to 100 mL								

Note: formulation was determined on weight basis not on volume. The % of drug was calculated and added on the weight basis of the polymer so as to ensure that 1 gm of formulation will contain 10 mg of drug.

**Table 4:** FTIR spectrum of timolol maleate.

Sr. number	Bond	Range (1/cm)	Observed peaks cm^−1^
1	N-H	3500–3300	3350.73
2	O-H	3700–3500	3185.83
3	C=N	2260–2210	2212.92
4	-CH_3_	2960–2850	2938.98
5	C=O	1740–1720	1714.41
6	N-S	910–900	857.20
7	C=C	1584	1568

**Table 5:** Mucoadhesive strength of batch f1–f9.

Formulation	Mucoadhesive strength (dyne/cm^2^)
f1	1172.42 ± 34.58
f2	1414.90 ± 89.25
f3	1845.39 ± 92.14
f4	1290.01 ± 54.12
f5	1469.17 ± 98.14
f6	2087.82 ± 58.36
f7	1814.90 ± 75.19
f8	1945.39 ± 84.15
f9	2483.62 ± 39.54

**Table 6:** ANOVA analysis.

Sr. number	Response model	Sum of squares	Df	Mean square	*F* value	*P* value	*R* ^2^	Adequate precision
1	Drug release at 480 min	1694.17	5	338.83	68.67	<0.0001 significant	0.9717	25.55
2	Gelation temperature	43.30	5	8.66	73.26	<0.0001 significant	0.9734	24.752

**Table 7:** Comparison of predicted and actual values of % drug release.

Polymers	Coded levels	Actual levels	Response	% drug release	Gelation temperature
			Predicted value	78.38	32.58
			Observed value	93.57	35
HPMC	0	0.8	Standard deviation	2.2213	0.3438
Poloxamer 407	0	46	Standard error mean	2.43	0.38

**Table 8:** Flux of *in situ* nasal gel.

Formulation code	Average flux cellophane membrane *µ*g/cm^2^/min	Average flux egg membrane *µ*g/cm^2^/min	Average flux nasal mucosa membrane *µ*g/cm^2^/min
f1	0.21	0.30	0.21
f4	0.20	0.25	0.22
